# Role of Circulating Tumor DNA Among Patients with Colorectal Peritoneal Metastases

**DOI:** 10.1007/s12029-023-00959-8

**Published:** 2023-07-12

**Authors:** Joel M. Baumgartner, Gregory P. Botta

**Affiliations:** 1 Division of Surgical Oncology, Department of Surgery, University of California, San Diego, La Jolla, CA, USA; 2 Division of Hematology-Oncology, Department of Medicine, University of California, San Diego, La Jolla, CA, USA

**Keywords:** ctDNA, Colorectal cancer, Peritoneal metastases

## Abstract

**Purpose:**

This was a review of circulating tumor DNA (ctDNA) in patients with peritoneal metastases from colorectal cancer.

**Methods:**

We searched the PubMed database for studies reporting detection of ctDNA in patients with colorectal cancer (CRC) and with peritoneal metastases (PM) from colorectal cancer (CRPM). We extracted data on the population included, number of subjects, study design, type of ctDNA assay used and schedule, and the major findings from these publications.

**Results:**

We identified 13 studies for review investigating ctDNA, using a variety of ctDNA assays, among 1787 patients with CRC without PM, as well as four eligible published and one unpublished (in press) studies, which included 255 patients with PM from any primary site and 61 patients with CRPM. Among the 13 studies investigating ctDNA among CRC without PM, posttreatment surveillance ctDNA was associated with recurrence and was generally more sensitive than imaging or tumor markers. Among the five studies including patients with PM, ctDNA was not universally able to detect the presence of PM, but when present, ctDNA predicted worse outcomes.

**Conclusion:**

Circulating-tumor DNA is a potentially useful surveillance tool for patients with CRC. However, the sensitivity of ctDNA to detect CRPM is variable and warrants further inquiry.

## Introduction

Colorectal cancer (CRC) is currently the third most common cancer in the USA with an annual incidence of approximately 150,000 [1]. It is the second most common cause of cancer-related deaths, contributing to approximately 52,000 annual deaths in the USA [1]. The peritoneum is a common site of metastasis, occurring in approximately 15% of patients with colorectal cancer [2]. The prognosis for patients with colorectal peritoneal metastases (CRPM) is poor, with a median survival of 12–16 months with systemic chemotherapy alone [3] and 42 months with surgical management in patients with isolated CRPM [4]. Visualization of CRPM is challenging with current imaging techniques, including computed tomography (CT), magnetic resonance imaging (MRI), and positron emission tomography (PET), which collectively have approximately an 85% sensitivity to detect peritoneal metastases (PM) [5]. As such, there remains significant treatment and diagnostic challenges with CRPM.

Cell-free DNA (cfDNA) is shed from both normal and diseased cells, most commonly those of hematopoietic origin. Furthermore, cfDNA is found in low concentrations within the bloodstream (1–10 ng/mL) and rapidly cleared by the liver (2.5-h half-life). DNA fragments released from tumor cells as circulating tumor DNA (ctDNA) are detectable in the bloodstream and comprise less than 0.1–10% of all circulating cfDNA [6]. Circulating tumor DNA is an emerging clinical tool for blood-based cancer screening, diagnosis, treatment guidance, and posttreatment surveillance [7].

Circulating tumor DNA assays vary by commercial availability, with each test having different gene numbers and types (somatic versus epigenetic) of alterations detected. A key difference is whether the analyzed genetic alterations are tumor tissue informed (defined from a patient’s tumor specimen) or tissue agnostic (extracted from a standardized alteration panel and gene methylation pattern) [7]. These variables considerably affect the intended use, sensitivity, and turnaround time of the ctDNA assay. Circulating tumor DNA has been investigated in multiple primary tumor types at multiple disease stages for the purpose of screening, detecting recurrence, surveying therapeutic response or resistance, and molecular profiling [8]. We sought to review the literature of ctDNA studies in CRC and in CRPM, specifically, as well as offer future directions for additional study.

## Methods

We performed a literature review by conducting a comprehensive search in PubMed/MEDLINE (through January 2023) using the following keywords: “circulating tumor DNA,” “ctDNA,” “colorectal cancer,” “peritoneal metastasis,” and “peritoneal carcinomatosis.” We included human studies published in English which analyzed ctDNA in CRC or peritoneal metastases (primarily CRPM) for surveillance or treatment guidance. Data extracted included the study population (stage of disease, proportion of patients with colorectal cancer in studies of peritoneal metastases), number of subjects, study design, type of ctDNA assay used and schedule, and the major findings from these publications.

### Circulating Tumor DNA in Colorectal Cancer

The feasibility of ctDNA detection as a CRC tumor-burden assessment was first demonstrated by Diehl et al. in 2008 [9]. This study analyzed serial plasma samples for ctDNA in 18 subjects with stages II–IV colorectal cancer undergoing therapy and found detectable ctDNA was more highly associated with recurrence after surgical resection compared to blood carcinoembryonic antigen (CEA). A number of subsequent studies have further analyzed the use of ctDNA assays in colorectal cancer, primarily assessing for minimal/molecular residual disease (MRD) to determine the necessity of adjuvant therapy and predict risk of recurrence after definitive therapy.

Several studies have been performed by Tie et al., using a ctDNA assay informed by the single tumor tissue alteration with the highest mean allele fraction (MAF, Safe-SeqS assay). This group initially demonstrated feasibility of ctDNA detection among 53 patients with metastatic CRC, and they found correlation of ctDNA levels with radiographic response and recurrence [10]. A subsequent study among 230 stage II colon cancer patients who underwent resection found 7.9% had detectable ctDNA postoperatively. Of those patients with positive ctDNA, 79% recurred versus only 9.8% of patients with negative ctDNA [11]. They also found an association between ctDNA detection and recurrence-free survival (RFS; *HR* 11, 95% *CI*, 1.8–68) among patients who received adjuvant chemotherapy. Another study among 159 patients with locally advanced rectal cancers found detection of ctDNA either post-chemoradiotherapy or post-resection was associated with worse RFS (*HR* 6.6, 95% *CI*, 2.6–17 and *HR* 13.0, 95% *CI*, 5.5–31, respectively) [12]. The 3-year RFS was 33% for patients with detectable postoperative ctDNA vs. 87% for postoperative ctDNA-negative patients. Furthermore, postoperative ctDNA status was an independent predictor of RFS on multivariate analysis. A stage III colon cancer study of 96 patients found an association between detectable postoperative ctDNA and post-adjuvant chemotherapy ctDNA and a shorter RFS (*HR* 3.8, 95% *CI*, 2.4–21.0 and *HR* 6.8, 95% *CI* 11.0–157.0, respectively). And again, postoperative ctDNA status was an independent predictor of RFS on multivariate analysis [13]. When evaluating the oligometastatic population, 54 patients undergoing resection for colorectal liver metastases found a ctDNA detection rate of 85% prior to surgery and 24% postoperatively [14]. Positive postoperative ctDNA was associated with shorter RFS (*HR* 6.3, 95% *CI*, 2.6–15.2) and OS (*HR* 4.2, 95% *CI*, 1.5–11.8) than those with negative postoperative ctDNA. End-of-treatment (postoperative and post-adjuvant therapy) ctDNA positivity had a 0% 5-year RFS versus 75.6% among those with negative end-of-treatment ctDNA (*HR* 14.9, 95% *CI*, 4.9–44.7).

Multiple additional single-arm studies have been performed investigating ctDNA for prognosis and surveillance in CRC. A Danish study of 130 patients with stages I-III CRC employing the tumor-informed ctDNA assay (Signatera) found (A) immediately postoperative, (B) post-adjuvant chemotherapy, and (C) surveillance detectable ctDNA was associated with higher rates of recurrence (*HR* 7.2, 95% *CI*, 2.7–19.0; *HR* 17.5, 95% *CI*, 5.4–56.5; and *HR* 43.5, 95% *CI* 9.8–193.5, respectively). Similar to previous studies, ctDNA remained independently associated with recurrence on multivariate analysis [15]. For those with radiographic recurrence, ctDNA detection preceded imaging findings of recurrence by a median of 8.7 months. A different study utilizing a tumor-informed orthogonal digital droplet PCR (ddPCR) ctDNA assay also found postoperative and surveillance ctDNA positivity were associated with disease-free survival (DFS; *HR* 6.96, 95% *CI* 2.57–18.91 and *HR* 8.03, 95% *CI* 1.79–35.98, respectively), and postoperative and serial ctDNA positivity remained a significant risk factor for DFS on multivariate analysis [16]. In the ddPCR ctDNA analysis, ctDNA detection preceded radiologic relapse by a median of 11.5 months. A second study using the ddPCR ctDNA assay among 29 patients with stages II–III rectal cancer found postoperative ctDNA positivity was also associated with PFS (*HR* 11.56, *p* = 0.007) [17].

In a study of 103 patients with stages I–IV CRC, a plasma-only (tumor tissue-agnostic) ctDNA assay incorporating genomic and epigenomic alterations (Guardant Reveal) was investigated [18]. Among patients with at least 1 year follow-up and with ctDNA-positive post-definitive treatment (postoperative and post-adjuvant therapy, if given), 100% recurred versus 24% of those with negative post-definitive treatment ctDNA (*HR* 11.2, *p* < 0.0001). The sensitivity of ctDNA to detect recurrence increased when longitudinal surveillance specimens were included (55.6 to 69.0%). A more recent study among 112 patients with metastatic, KRAS-mutant CRC compared two ctDNA assays: the Signatera tumor-informed assay and a KRAS alteration ctDNA panel [19]. Positive detection of postoperative ctDNA by the tumor-informed assay was associated with worse DFS (*HR* 5.8, 95% *CI* 3.5–9.7) and OS (*HR* 16.0, 95% *CI* 3.9–68) than those with undetectable postoperative ctDNA. Circulating tumor DNA remained a significant risk factor for worse DFS on multivariate analysis (*HR* 5.78, 95% *CI* 3.34–10.0). No stratification by metastatic site was performed. When comparing the two ctDNA assays, investigators found 44.5% discordance, with all discordant cases having undetectable ctDNA by KRAS panel but positive by the 16-alteration tumor-informed panel. Furthermore, 91.6% of the discordant cases recurred, suggesting higher sensitivity of the 16 gene tumor-informed ctDNA assay than the KRAS alteration assay. Another multicenter study using the 16 gene tumor-informed ctDNA assay in 168 patients with stage III CRC found postoperative (*HR* 7.0, 95% *CI* 3.7–13.5) and post-adjuvant therapy surveillance (*HR* 50.76, 95% *CI* 15.4–167) ctDNA detection predicted RFS [20]. The only patients with detectable postoperative ctDNA who did not recur were those who cleared ctDNA permanently after adjuvant therapy. The lead time for ctDNA versus radiologic detection of recurrence was a median of 9.8 months.

A recent retrospective study among 48 patients with stages II–IV CRC compared the surveillance sensitivity of the 16 gene tumor-informed ctDNA assay to imaging plus traditional carcinoembryonic antigen (CEA) for recurrence and found that ctDNA did not improve the sensitivity compared to imaging plus CEA (53.3% vs. 60.0% sensitivity, *p* > 0.99). Furthermore, ctDNA did not detect recurrence earlier than imaging plus CEA (14.3 months vs. 15.0 months) [21]. These findings, from a relatively small retrospective and single-center study, are in contrast to the studies above but highlight the notion that surveillance ctDNA in CRC may not be an actionable finding beyond imaging and standard of care blood biomarkers alone [22].

There has been one published prospective randomized controlled trial investigating ctDNA-guided adjuvant therapy in CRC [23]. This study, performed by Tie et al., randomized 455 patients with stage 2 CRC to ctDNA-guided adjuvant therapy (using the Safe-SeqS tumor-informed alteration assay) versus standard of care (clinicopathologic)-guided adjuvant therapy. They found 15% of patients in the ctDNA arm had detectable ctDNA and received adjuvant chemotherapy versus 28% receiving adjuvant therapy in the standard-management group. The ctDNA-guided group had a noninferior 2-year RFS compared to the standard-management group (93.5% v. 92.4%, absolute difference 1.1%, 95% *CI* − 4.1–6.2). The 3-year RFS was 86.4% among ctDNA-positive patients who received chemotherapy and 92.5% among those without detectable ctDNA who did not receive adjuvant chemotherapy Table 1 .

**Table 1:** Circulating tumor DNA studies in colorectal cancer

**Study**	**Population**	***n***	**Design**	**ctDNA assay**	**ctDNA assay schedule**	**Major findings**
Tie et al. (2015) [10]	Stage IV CRC	53	Single arm	Single tumor-informed alteration	Pretreatment, 3-day post-treatment, pre-cycle 2	ctDNA decrease correlated with radiographic response and PFS
Tie et al. (2016) [11]	Stage II CC	230	Single arm	Single tumor-informed alteration	4–10 weeks, Q3 months × 2 years postoperative	Post-op ctDNA associated with recurrence
Reinert et al. (2019) [15]	Stages I–III CRC	130	Single arm	16 gene tumor-informed alterations	Preoperative, POD 30, and Q3 months × 3 years	Postop, post-adjuvant chemo, and surveillance ctDNA associated with RFS
Tarazona et al. (2019) [16]	Stages I–III CC	150	Single arm	1–2 tumor-informed alterations	Preoperative, 6–8 weeks postoperative, Q4 months × 5 years	Postop and surveillance ctDNA associated with DFS
Tie et al. (2019) [12]	T3–4 and/or N + RC	159	Single arm	Single tumor-informed alteration	Pretreatment, 4–6 week postchemoradiotherapy, 4–10 weeks postoperative	Post-chemoradiotherapy or post-op ctDNA associated with RFS, even on MVA
Tie et al. (2019) [13]	Stage III CC	96	Single arm	Single tumor-informed alteration	4–10 weeks, within 6 weeks after adjuvant chemotherapy	Post-op and post-adjuvant chemo ctDNA associated with RFS
Loupakis et al. (2021) [19]	Stage IV CRC	112	Single arm	16 gene tumor-informed alterations and KRAS panel	Postoperative, after radiologic recurrence or f/u	Post-op ctDNA associated with DFS, possibly higher sensitivity of 16 alteration tumor-informed assay than the KRAS assay
McDuff et al. (2021) [17]	Stages II–III RC	29	Single arm	1–2 tumor-informed alterations	Preoperative, 1–5 months postoperative	Post-op ctDNA-associated RFS
Parikh et al. (2021) [18]	Stages I–IV CRC	103	Single arm	Plasma-only genomic and epigenomic alterations	Preoperative, 4 weeks postoperative, 4 weeks post-adjuvant treatment, various surveillance timepoints	Post-definitive treatment ctDNA associated with recurrence
Tie et al. (2021) [14]	CRLM	54	Single arm	Single tumor-informed alteration	Preoperative, pre-cycle neoadjuvant, 4–10 weeks postoperative, posttreatment, Q3 months × 1 year, Q6 months × 1 follow-up	Post-op ctDNA associated with RFS and OS
Fakih et al. (2022) [21]	Stages II–IV CRC	48	Single arm	16 gene tumor-informed alterations	Postoperative Q3 months × 2 years, Q6 months × 3 years	No difference in ctDNA sensitivity for recurrence than imaging
Henriksen et al. (2022) [20]	Stage III CRC	168	Single arm	16 gene tumor-informed alterations	Postoperative, Q3 months after adjuvant therapy	Post-op and surveillance ctDNA associated with RFS
Tie et al. (2022) [23]	Stage II CC	455	Double-arm RCT	Single tumor-informed alteration	4 weeks and 7 weeks postoperative	Non-inferior RFS in ctDNA-guided adjuvant therapy vs. clinicopathologic-guided adjuvant therapy

*CRC* colorectal cancer, *CC* colon cancer, *RC* rectal cancer, *CRLM* colorectal liver metastases, *wks* weeks, *mos* months, *yrs* years, *HR* hazard ratio, *PFS* progression-free survival, *RFS* recurrence-free survival, *OS* overall survival, *POD* postoperative day, *KRAS* Kirsten rat sarcoma virus, *RCT* randomized controlled trial

### Circulating Tumor DNA from Peritoneal Metastases

The detectability of ctDNA from primary and metastatic peritoneal sites remains challenging. One challenge is that ctDNA from peritoneal disease may not enter the vasculature, limiting blood-based gene assays. The second issue is that peritoneal metastases may generate lower levels of ctDNA than other metastatic sites [6]. The rationale for lower detection levels of ctDNA from peritoneal metastases are uncertain but may include lower and more disorganized vascular density, inhibition by the plasma-peritoneal barrier, and mucin production disrupting ctDNA vascular entry [24–26].

We have previously investigated perioperative ctDNA among patients undergoing surgery for colorectal peritoneal metastases (CRPM) and found approximately 63% had detectable preoperative and postoperative ctDNA, using a 73-gene tissue-agnostic panel (Guardant360). Importantly, those with high levels of preoperative or postoperative detectable ctDNA had worse PFS [27, 28]. There was high concordance between preoperative ctDNA and tissue DNA genomic alterations [29].

Another study investigated ctDNA analysis among 30 patients undergoing curative-intent cytoreductive surgery and hyperthermic intraperitoneal chemotherapy (CRS-HIPEC) for CRPM [30]. They used a 3-gene alteration tumor-informed ctDNA assay and found that 33% of patients had detectable ctDNA preoperatively which was associated with reduced DFS. In the post-CRS-HIPEC cases, only 4% (one patient) had detectable ctDNA, and this patient had radiographic distant recurrence 7 months later.

A larger retrospective study of 279 patients with stages II–IV gastrointestinal cancers investigated pretreatment ctDNA using a tumor-agnostic panel (Gaurdant360) and found that higher rates of detectable ctDNA correlated with higher stages of disease. There was a lower maximum variant allele frequency (mVAF) in those with peritoneal metastases than in those with non-peritoneal metastases (although the peritoneal metastasis patients had 72.1% ctDNA detection rate and the results were not stratified by primary tumor site) [31]. The authors concluded that “caution is warranted when interpreting ctDNA results” from patients with peritoneal metastases.

Recently, Singh et al. retrospectively evaluated the 16 gene tumor-informed assay in the clinical management of 13 patients with radiographically occult but pathologically confirmed peritoneal metastases (eight appendiceal and five gastric cancers) [32]. Detectable tumor-informed ctDNA was identified in 62% (8/13) of patients: three appendiceal (50% of cases) and five gastric (100% of cases) cancers. Three appendiceal patients had negative ctDNA, but detectable occult disease on laparoscopic analysis and two appendiceal cancer patients were unable to create a baseline test due to insufficient tissue. Even with low detectable ctDNA within the blood, disease burden correlated with longitudinal ctDNA analysis. Furthermore, nine of 13 patients had no other blood biomarker to follow their disease, whereas ctDNA was informative.

### Future Directions of ctDNA in CRPM

Circulating tumor DNA has potential for risk stratification, surveillance for recurrence, and identification of actionable genetic alterations in patients with cancer. Patients with CRC have shown a significant association with ctDNA and RFS across multiple studies. It typically precedes imaging findings of recurrence by 8–12 months. A major unanswered question, however, is whether earlier detection of recurrence by ctDNA improves outcomes and if interventions should occur at ctDNA detection. There are cases of patients with increasing ctDNA without radiographic disease of progression that later have a reduction of ctDNA without intervention. It is unknown if a flood of cfDNA from another source mimics the ctDNA of the tumor, if a tumor cell indeed developed but immune surveillance responded appropriately, or if the assay resulted a true–false positive. Although not proven in solid tumor clinical trials, there is a rationale for focusing treatment efforts on minimal residual disease to improve oncologic outcomes [33]. Radiologic occult disease is of highest concern, especially in terms of peritoneal dissemination without definable or measurable masses. The disease burden within the abdomen can cause obstruction complications without early intervention. Furthermore, it is theorized that earlier disease stages may have more intact immune surveillance and potential responses versus those with later stage disease [34, 35]. As such, with less toxic and more effective treatments in low-burden disease states (i.e., immunotherapy), attempting treatment at the earliest sign of progression with ctDNA may provide improved quality of life and progression-free survival above lead time bias. Already, ctDNA has been shown to be predictive of immunotherapy responses in patients with solid tumors before radiologic response [36]. Withholding adjuvant therapy in patients who are ctDNA-negative post-resection in non-metastatic, low-risk CRC does not appear to cause harm [23], and additional studies are underway to determine how ctDNA might alter adjuvant treatment decisions in CRC [37].

Use of ctDNA in patients with CRPM for risk stratification has not been well-studied. Questions remain regarding the sensitivity of ctDNA assays in these patients. While some assays allow for identification of actionable genetic alterations to guide systemic therapy, this may be better determined through tumor tissue analysis than blood-based methods. Considering its paucity in the bloodstream, tumor-informed, multiple gene ctDNA assays provide higher sensitivity for peritoneal disease versus single, fixed, or droplet PCR ctDNA assays. Utilization of ctDNA for surveillance in patients with CRPM remains uncertain, and further research is needed to compare sensitivity of various ctDNA assays, as well as to determine the optimal timing and frequency of ctDNA analysis in this population. Furthermore, if blood is not an adequate milieu to evaluate ctDNA, peritoneal washings or ascites may be a more accurate source of ctDNA in CRPM patients, although this is not always available. Similar to nonmetastatic CRC, it also is critical to determine whether earlier detection of recurrence or progression by ctDNA and subsequent interventions in CRPM patients improves oncologic outcomes and quality of life.
